# Perceived Access to Contraception via Telemedicine Among Young Adults: Inequities by Food and Housing Insecurity

**DOI:** 10.1007/s11606-022-07669-0

**Published:** 2022-06-03

**Authors:** Jennifer Yarger, Kristine Hopkins, Sarah Elmes, Irene Rossetto, Stephanie De La Melena, Charles E. McCulloch, Kari White, Cynthia C. Harper

**Affiliations:** 1 Bixby Center for Global Reproductive Health, Department of Obstetrics, Gynecology and Reproductive Sciences, University of California, San Francisco, 550 16th Street, San Francisco, CA 94158 USA; 2 Population Research Center, University of Texas at Austin, Austin, TX USA; 3 Department of Epidemiology and Biostatistics, University of California, San Francisco, San Francisco, CA USA

**Keywords:** telemedicine, contraception, housing insecurity, food insecurity, access to care, COVID-19

## Abstract

**Background:**

Telemedicine expanded rapidly during the COVID-19 pandemic, including for contraceptive services. Data are needed to understand whether young people can access telemedicine for contraception, especially in underserved populations.

**Objective:**

To compare young people’s perceived access to telemedicine visits for contraception during the COVID-19 pandemic by food and housing insecurity.

**Design:**

Supplementary study to a cluster randomized controlled trial in 25 community colleges in California and Texas. Online surveys were administered May 2020 to April 2021. Mixed-effects logistic regression models with random effects for site were used to examine differences in access to contraception through telemedicine by food and housing insecurity status, controlling for key sociodemographic characteristics, including race/ethnicity, non-English primary language, health insurance status, and state of residence, and contraceptive method used.

**Participants:**

1,414 individuals assigned female at birth aged 18–28.

**Main Measures:**

Survey measures were used to capture how difficult it would be for a participant to have a telemedicine visit (phone or video) for contraception.

**Key Results:**

Twenty-nine percent of participants were food insecure, and 15% were housing insecure. Nearly a quarter (24%) stated that it would be difficult to have a phone or video visit for contraception. After accounting for sociodemographic factors and type of method used, food insecure (adjusted odds ratio [aOR], 2.17; 95% confidence interval [CI], 1.62–2.91) and housing insecure (aOR, 1.62; 95% CI, 1.13–2.33) participants were significantly more likely to report that it would be difficult to use telemedicine for contraception during the pandemic.

**Conclusions:**

Underserved patients are those who could benefit most from the expansion of telemedicine services, yet our findings show that young people experiencing basic needs insecurity perceive the greatest difficulty accessing these services for essential reproductive care.

**Trial Registration:**

ClinicalTrials.gov Identifier: NCT03519685

## INTRODUCTION

The coronavirus disease 19 (COVID-19) pandemic has motivated providers to expand telemedicine services in order to minimize in-person encounters, including for contraceptive care.^1,2^ One study found that more than half of family planning providers in the USA conducted telemedicine visits for contraception during the pandemic, compared to just 17% before the pandemic.^3^ Using communication technologies, telemedicine offers a safe and effective way to support contraceptive initiation, adherence, and continuation.^4–6^

Telemedicine can help increase access to care, particularly for rural and underserved populations, by removing the need for transportation, childcare, and additional time for in-person office visits. However, research has found that underserved patient populations, including Medicaid beneficiaries and low-income and rural populations, are less likely to use telemedicine than other populations.^7–9^ Commentaries have cautioned that the most vulnerable populations may face overlapping barriers to telemedicine during the COVID-19 pandemic, including limited access to technology, low telehealth literacy, and financial constraints.^10–13^ Evidence is needed to understand barriers to accessing telemedicine during the pandemic, including for young people.

Little is known about access to telemedicine visits specifically for contraceptive services. Researchers have speculated that telemedicine growth during the pandemic may increase access to contraceptives and other services differentially and heighten service gaps for marginalized young people.^14^ A national study of 18–49-year-olds found that one-quarter of pill users had switched to a telemedicine appointment to have their prescription refilled, but the study did not examine disparities in telemedicine access for contraception.^15^ A small New York City study conducted during the pandemic found that people were satisfied with telemedicine visits for contraceptive counseling, although the study included only patients who utilized telemedicine, missing those who might face substantial access barriers.^16^ One study examined disparities in telemedicine use for family planning services and found that Black and multi-racial patients were less likely to use telemedicine services compared to in-clinic care, although the study was also limited to a patient population, who had received care.^17^ It is important to also consider evidence from non-clinic populations, including schools, to capture experiences of young people who may be in need of care but unable to access it.

The current study examines perceived access to telemedicine for contraception by food and housing insecurity status among young people in a community college study. Basic needs insecurity historically has been high among students attending community college,^18–20^ and it has grown for all populations during the pandemic due to widespread job and income loss.^21^ Previous research has demonstrated that food and housing insecurity are associated with postponing medical care and medications.^22^ Informed by the social determinants of health framework,^23^ we hypothesized that young adults who were food and housing insecure would perceive less access to telemedicine visits for contraception compared to those who were food and housing secure. We considered other important contextual sociodemographic factors including racial/ethnic, language, and insurance barriers to understand how food and housing insecurity may add even more challenges to accessing care for young people.

## METHODS

This study is supplementary to an ongoing randomized controlled trial of an intervention to increase contraceptive education and access among young people attending community college. The overall study was launched in April 2018. Participants were recruited at 25 community college sites in California and Texas, two of the most populous and racially/ethnically diverse states. Among women who likely need public support for contraceptive services, more than one in five live in California or Texas.^24^ Participants were eligible if they were aged 18–25, assigned female at birth, spoke English, had vaginal sex with a male partner in the last year, not currently pregnant or wanting to become pregnant, and students at the participating community college site at enrollment. Participants completed online surveys at baseline, every 3 months for 1 year, and every 6 months thereafter for reproductive health, educational, and economic outcomes. Survey reminders were sent to participants via email, text message, social media direct message, or phone call.

In May 2020, we added a series of items to each baseline and follow-up survey to assess the impact of the COVID-19 pandemic on young people’s sexual and reproductive health, health access, education, and economic well-being, and their access to telemedicine. The current analyses used surveys administered May 2020 to April 2021. If a participant completed more than one survey during this period, we included data from the last survey completed (*N*=1,414). Most surveys (85%) were completed between January and April 2021.

This study was approved by the Institutional Review Boards (IRBs) at the University of California, San Francisco and the University of Texas at Austin; participating college sites either approved the study with their IRB or used the corresponding state university’s IRB approval. In order to integrate community feedback into the research process, we gathered input from members of the participating college communities at different stages. We consulted our community advisory board prior to and during the study and conducted interviews with students, faculty, and staff about the research process. For example, we asked students how they felt about research on their campus and what was the best way to engage students, and asked them to review study flyers to ensure that the language and images were inclusive of diverse populations. Participants received a $50 electronic gift card following study enrollment and a $20 gift card after each follow-up survey, except for the 12-month survey after which they received a $30 gift card.

### Measures

#### Perceived Difficulty Having a Telemedicine Visit for Contraception

The primary study outcome was perceived difficulty having a telemedicine visit for contraception. The survey items were “How difficult would it be for you to have a **phone visit** to get a birth control method?” and “How difficult would it be for you to have a **video visit** to get a birth control method?” (very difficult, difficult, neutral, easy, very easy). From these questions, we created a dichotomous variable equal to 1 if the participant answered “very difficult” or “difficult” to either question.

We also assessed perceived barriers to phone and video visits for contraception, including the following: “My doctor/clinic does not offer (phone/video) visits”; “My health insurance would not cover a (phone/video) visit”; “I would have to get my birth control method in person”; “I would not have (a phone/the necessary electronic devices)”; “I would not have a reliable Internet connection”; “I would not know how to do a (phone/video) visit”; “I would not feel comfortable with a (phone/video) visit”; “I would not have enough privacy at home”; and “I would prefer an in-person visit.” All participants were asked about perceived barriers to telemedicine (yes, no, don’t know), regardless of whether they have used telemedicine. We created dichotomous variables equal to 1 if the participant answered “yes” to each perceived barrier.

#### Food Insecurity

Participants were asked an item adapted from the U.S. Department of Agriculture household food security module, which assesses the least severe condition that indicates food insecurity.^25,26^ Participants were asked how often their household worried whether their food would run out before they got money to buy more in the last month (often true, sometimes true, or never true). We created a dichotomous measure of whether participants experienced food insecurity (1 = often true/sometimes true, 0 = never true).

#### Housing Insecurity

Two questions were used to assess participants’ housing insecurity. “In the past year, were you homeless at any time? (*Note: This includes living in a car, on the street, or staying in a homeless or temporary shelter*.)” (yes, no) and “In the past year, did you face other problems with housing instability? (*Note: This may include having to move many times.*)” (yes, no). Participants were coded as housing insecure if they responded affirmatively to either question.

#### Covariates

We collected sociodemographic characteristics that have been associated with use of telemedicine during the pandemic^8,9^: age (18–19, 20–21, 22 years or older), self-reported race/ethnicity (Hispanic, White non-Hispanic, Asian/Pacific Islander non-Hispanic, Black non-Hispanic, American Indian/other/multi-racial non-Hispanic), language spoken at home (English, language other than English), and health insurance status (uninsured, insured or don’t know). We also controlled for whether participants were living with a parent, due to potential privacy concerns about telemedicine visits, state of residence (California, Texas), and contraceptive method used (the pill/patch/ring/emergency contraceptive pill, condom, injectable, intra-uterine device (IUD)/subdermal implant, other/none).

### Statistical Analysis

We compared participant characteristics by food and housing insecurity using univariate logistic regression for dichotomous characteristics and multinomial logistic regression for those with more than two categories, with cluster robust standard errors. To compare differences in perceived difficulty of having a telemedicine visit for contraception by basic needs insecurity, we used multivariable mixed-effects logistic regression models with random effects for site. We ran two separate models: one with food insecurity as the primary independent variable and another with housing insecurity. All models controlled for age, race/ethnicity, language spoken at home, health insurance status, living with parents, state of residence, and type of method used. Finally, we described perceived barriers to phone and video visits for contraception and used univariate logistic regression with cluster robust standard errors to compare perceived barriers by food and housing insecurity. We used listwise deletion to handle the missing data (<5%). Analyses were conducted in Stata version 16 and significance levels reported at *p* < 0.05.

## RESULTS

### Sample Characteristics

The mean age of the participants was 20.5 years (sd 1.7 years). The sample was racially and ethnically diverse with 58% Hispanic, and among the non-Hispanic participants, 21% White, 9% Asian/Pacific Islander, 6% Black, and 6% American Indian/other/multi-racial (Table 1). Half of participants spoke a language other than English at home (50%), and 62% were living with a parent. Most (77%) were still enrolled in college at the time of the survey. One in five (21%) were receiving public assistance, and 16% were uninsured. Eight percent of participants had children. Most participants were currently sexually active (87%) and currently using a contraceptive method (78%).

**Table 1:** Participant Characteristics, by Food and Housing Insecurity Status (*N*=1,414)

Characteristics	Total, %	Food insecure (*n*=404; 29%), %	Housing insecure (*n*=216; 15%), %
Age			
18–19 years	25	23	16*
20–21 years (ref)	58	53	60
22 years or older	17	24***	24
Race/ethnicity			
Hispanic (ref)	58	60	54
White non-Hispanic	21	18	23
Asian/Pacific Islander non-Hispanic	9	9	7
Black non-Hispanic	6	8	8
American Indian/other/multi-racial non-Hispanic	6	5	8
Speaks non-English language at home	50	51	43*
Lives with parent	62	47***	40***
Enrolled in college	77	71***	68***
Employment status			
Employed full-time	23	27*	29*
Employed part-time (ref)	42	35	34
Not employed	35	38*	37
Receives public assistance	21	32***	36***
Uninsured	16	20	21
Has child(ren)	8	13***	11
Had vaginal sex in the past 3 months	87	90*	88
Type of method used			
IUD/implant	22	25*	24
Pill/patch/ring/emergency contraceptive pill (ref)	22	19	18
Condom	22	18	18
Injectable	2	2	2
Other	10	10	12
None	22	26**	25*
State of residence			
California	71	71	75
Texas	29	29	25

*Note*: *p < .05, **p < .01, ***p <.001. Univariate logistic (for dichotomous characteristics) and multinomial logistic (for characteristics with more than two response categories) regression models with cluster robust standard errors were used to compare the food insecure to food secure and to compare the housing insecure to housing secure

Over one-quarter of participants reported food insecurity (29%), and 15% housing insecurity (Table 1). Food and housing insecurity were associated with several common factors capturing adversity, including receipt of public assistance. Of note, both food and housing insecurity were significantly associated with no longer being enrolled in school, working full-time, not living with parents, and no contraceptive method.

### Perceived Difficulty Accessing Telemedicine for Contraception

More than one in five participants (22%) reported that it would be difficult to have a telemedicine visit for contraception. In total, 7% of participants believed that both phone and video visits would be difficult, 10% believed that only video visits would be difficult, and 4% believed that only phone visits would be difficult.

Perceptions of access to telemedicine for contraception varied significantly by basic needs insecurity. Thirty percent of the food insecure and 27% of the housing insecure reported that it would be difficult to have a phone or video visit to get birth control, compared to 19% of the food secure (*p* < .001) and 21% of the housing secure (*p* = .032) (Table 2).

**Table 2:** Perceived Difficulty Accessing Telemedicine for Contraception, by Food Insecurity and Housing Insecurity Status (*N*=1,357)

Basic needs insecurity	Perceived difficulty having a telemedicine visit for birth control			
	*n*	%	Adjusted odds ratio*	95% confidence interval
*Model 1*				
Food				
Food insecure	119	30.1	2.17	1.62–2.91
Food secure	185	18.5	1.00 (Ref)	
*Model 2*				
Housing				
Housing insecure	58	27.4	1.62	1.13–2.33
Housing secure	246	20.8	1.00 (Ref)	

*Multivariable mixed-effects logistic regression models controlled for age, race/ethnicity, speaks non-English language at home, lives with parent, health insurance status, state of residence, type of method used, and included random effects for site

In the multivariable logistic regression models controlling for sociodemographic characteristics and type of method used, participants who were food insecure were significantly more likely to perceive access to telemedicine for contraception as difficult (adjusted odds ratio (aOR), 2.17; 95% confidence interval (CI), 1.62–2.91) than those who were food secure. Participants also were more likely to perceive access to telemedicine for contraception as difficult if they were experiencing housing insecurity (aOR, 1.62; 95% CI, 1.13–2.33).

As a sensitivity test, we also used multivariable mixed-effects ordered logistic regression to model the association between food and housing insecurity and perceived access to a telemedicine visit for contraception, using categorical variables with all five response options (very difficult, difficult, neutral, easy, very easy). The results also showed that food and housing insecurity were significantly associated with greater perceived difficulty in accessing telemedicine for contraception.

### Perceived Barriers to Accessing Telemedicine for Contraception

The most common perceived barriers to telemedicine visits for contraception were needing to have an in-person visit for their method (43% for phone, 41% for video), lack of privacy at home (35% for phone, 35% for video), and lack of comfort with telemedicine (24% for phone, 26% for video) (Table 3). Technology-related perceived barriers to telemedicine for contraception were less common, although still a challenge for many participants. Most stated that they would prefer an in-person visit over a phone (73%) or video visit (71%) for contraception.

**Table 3:** Perceived Barriers to Telemedicine Visits for Contraception, by Food and Housing Insecurity Status (*N*=1,401)

	Phone visit for contraception			Video visit for contraception		
	Total, %	Food insecure (*n*=394), %	Housing insecure (*n*=213), %	Total, %	Food insecure (*n*=394), %	Housing insecure (*n*=213), %
***Perceived barrier***						
I would have to get my birth control method in person	43.2	49.7**	45.7	40.5	46.6**	38.9
I would not have enough privacy at home	35.3	38.5	37.1	35.4	40.3**	36.0
I would not feel comfortable with a (phone/video) visit	24.2	30.5***	24.1	26.4	33.1***	27.5
I would not know how to do a (phone/video) visit	16.6	19.6	19.0	15.9	20.2***	15.6
My doctor/clinic does not offer (phone/video) visits	11.5	13.0	11.8	8.7	10.2	10.3
My health insurance would not cover a (phone/video) visit	10.1	13.2*	12.7	8.7	11.5**	10.5
I would not have (a phone/the necessary electronic devices)	6.7	8.9	8.1	7.6	11.6**	11.0*
I would not have a reliable Internet connection				16.6	24.0***	19.4
***Preference***						
I would prefer an in-person visit	73.3	73.9	66.7**	70.7	72.0	68.1

*Note*: *p < .05, **p < .01, ***p <.001. Univariate logistic regression models with cluster robust standard errors were used to compare the food insecure to food secure and to compare the housing insecure to housing secure

Participants experiencing food insecurity were significantly more likely to report each of the perceived barriers to video visits for contraception than the food secure, except for their provider not offering telemedicine. When asked about perceived barriers to a phone visit for contraception, the food insecure were more likely to report that they would need to get their method in person, they would not feel comfortable with a phone visit, and their insurance would not cover it. The housing insecure were more likely to report that they would not have the necessary device for a video visit and that they would prefer an in-person visit over a phone visit.

Notably, many participants reported a lack of knowledge about telemedicine. In total, 17% believed that they would not know how to do a phone visit, and 16% would not know how to do a video visit. Moreover, “don’t know” was a common response when asked about each perceived barrier to telemedicine use (data not shown). About half did not know if their doctor/clinic offered video (51%) or phone visits (44%). More than one-third did not know if their health insurance would cover telemedicine (36% for phone visits, 41% for video visits). Twenty percent (for phone and for video) did not know if they would need to get their birth control method in person.

## DISCUSSION

### Principal Findings

Health care providers turned to telemedicine to an unprecedented degree to preserve access to reproductive health care during the COVID-19 pandemic.^27–29^ However, nearly one-quarter of young adults in this study perceived that it would be difficult for them to use telemedicine for contraception. Young adults who were food and housing insecure were particularly likely to perceive limited access to telemedicine for contraception. These findings are consistent with previous research that demonstrated an association between basic needs insecurity and difficulty accessing medical care and medication.^22^ These findings are also consistent with studies that found women who experienced poverty, income loss, or hunger reported impeded contraceptive access during the pandemic.^15,30,31^ While the shift to telemedicine helped to minimize COVID-19 exposure, it may have exacerbated existing inequities in access to contraceptive care.

Providers should take steps to help ensure equal access to telemedicine for contraception. Many young people expressed privacy concerns, which is consistent with qualitative research conducted pre-pandemic.^32,33^ Encouraging patients to wear headphones, using yes/no questions, and leveraging the chat function of video conferencing platforms are examples of strategies for addressing privacy concerns in telemedicine visits.^28^ Although young adults have been more likely to make video calls during the pandemic than other age groups,^34^ a quarter of young people in the study stated that they would not feel comfortable having a telemedicine visit for contraception. Research is needed to identify best practices for youth-friendly telemedicine visits, building on the body of literature measuring youth-friendly sexual and reproductive health services.^35^ At the same time, it is critical to give youth a choice between a telemedicine and in-person visit, respecting individual preferences and acknowledging disparities in access to telemedicine visits.

Many young people lacked basic knowledge about telemedicine for contraception. Outreach and education are needed to increase knowledge of telemedicine, including which contraceptive methods require an in-person visit, and to build health literacy skills that enable young people to access and understand information about telemedicine. Research increasingly highlights the need to develop health literacy among adults of all ages,^36,37^ including telehealth literacy.^10^ Interventions to increase telehealth literacy should partner with existing programs for food and housing insecurity, including those offered in colleges and universities.^38^ For example, campus food pantries can distribute printed educational materials about telemedicine services offered by the college health center or nearby community clinics.

### Limitations

This study has limitations. Although our sample was diverse in terms of race/ethnicity and socioeconomic characteristics, it is not generalizable to the population at large. Participants were largely community college students, a population who may have greater access to the Internet and electronic devices and therefore telemedicine than non-student populations. Nonetheless, our findings consistently showed economic disparities in young people’s perceptions of access to telemedicine for contraception. Another limitation is that most of the surveys were completed January to April 2021, and perceptions of telemedicine may have been different earlier or later in the pandemic as telemedicine use evolved. In addition, the survey questions did not measure perceptions of difficulty accessing in-person visits for contraception. Future research should compare perceived access to telemedicine and in-person visits. Finally, young people’s perceptions of access to telemedicine may differ from their actual access. However, perceptions are important in their own right, given the evidence that women who perceive difficulty in accessing health care may decide to delay or forgo needed care.^39,40^

### Implications for Public Health

This study presents evidence that adverse socioeconomic conditions are associated with less perceived access to telemedicine for essential sexual and reproductive health care. We found that young people experiencing food and housing insecurity were more likely to report that it would be difficult to have a telemedicine visit for contraception. Family planning providers in the USA have voiced concern that the shift to telemedicine visits could lead to an increase in health disparities for patients.^3^ More research is needed to examine whether disparities in telemedicine access contributed to reproductive health inequalities and whether they have worsened during the pandemic.

Telemedicine will likely continue to expand past the COVID-19 pandemic. A variety of strategies are needed to improve access to telemedicine, particularly for the most vulnerable populations, in order to reduce inequities in reproductive autonomy and health. As telemedicine evolves, it will be critical to engage young people in research outside clinic settings to identify the populations not being reached and the barriers they are facing.
